# Age differences in mental health literacy

**DOI:** 10.1186/1471-2458-8-125

**Published:** 2008-04-20

**Authors:** Louise Farrer, Liana Leach, Kathleen M Griffiths, Helen Christensen, Anthony F Jorm

**Affiliations:** 1 Centre for Mental Health Research, Australian National University, Canberra, ACT, 0200, Australia; 2 ORYGEN Research Centre, Department of Psychiatry, University of Melbourne, Locked Bag 10, Parkville, Victoria, 3052, Australia

## Abstract

**Background:**

The community's knowledge and beliefs about mental health problems, their risk factors, treatments and sources of help may vary as a function of age.

**Methods:**

Data were taken from an epidemiological survey conducted during 2003–2004 with a national clustered sample of Australian adults aged 18 years and over. Following the presentation of a vignette describing depression (n = 1001) or schizophrenia (n = 997), respondents were asked a series of questions relating to their knowledge and recognition of the disorder, beliefs about the helpfulness of treating professionals and medical, psychological and lifestyle treatments, and likely causes.

**Results:**

Participant age was coded into five categories and cross-tabulated with mental health literacy variables. Comparisons between age groups revealed that although older adults (70+ years) were poorer than younger age groups at correctly recognising depression and schizophrenia, young adults (18–24 years) were more likely to misidentify schizophrenia as depression. Differences were also observed between younger and older age groups in terms of beliefs about the helpfulness of certain treating professionals and medical and lifestyle treatments for depression and schizophrenia, and older respondents were more likely to believe that schizophrenia could be caused by character weakness.

**Conclusion:**

Differences in mental health literacy across the adult lifespan suggest that more specific, age appropriate messages about mental health are required for younger and older age groups. The tendency for young adults to 'over-identify' depression signals the need for awareness campaigns to focus on differentiation between mental disorders.

## Background

It is now well recognised that up to 70% of individuals with mental health disorders do not seek help. It has been argued that help seeking will improve with better recognition and labelling of mental disorders, increased understanding of the causes and treatments for mental health problems [1], reductions in stigma [2] and confidence and belief in the rationale for treatment approaches [3]. The advantages of early help seeking have been clearly articulated, with early help seeking providing the opportunity for early intervention and improved long-term outcomes for mental disorders [4]. While mental health literacy in the general community has received considerable research attention, the topic of age differences in this area is under investigated [5]. Such information may be particularly important in the development of mental health literacy campaigns that target different age groups.

Psychosis and depression often have onset during adolescence or early adulthood [6,7] and the rate of mental disorders in youth has been estimated to be between 20–27% [8]. This suggests that this age group is an important target for raising knowledge and awareness of mental health disorders. However, the mental health literacy of adolescents and young adults is not high. Previous research indicates that less than 50% of young people aged 12–25 years are able to identify depression correctly and only about a quarter are able to identify psychosis [9]. Slightly better rates were found in a smaller sample by Burns and Rapee [8]. These rates of recognition are much lower in young men than young women [10]. Nevertheless, there is evidence that mental health literacy among young people may be superior to that of older people [11,12]. Using a representative sample of South Australians, Fisher and Goldney found that older people (65–74 years) were poorer at correctly identifying depression than young adults (15–24 years) [11]. In addition, fewer people in the older age group recommended treatment for depression from a counsellor, telephone service or psychologist, in comparison to the younger age group, and more people in the older age group considered seeing a psychiatrist as unhelpful and a member of the clergy as helpful. A study conducted by Hasin and Link also found that older participants were less likely to characterise a vignette describing major depression as depressed, after controlling for sociodemographic factors [12]. Internationally, age differences have been investigated in relation to attitudes to depression [13], attitudes to mental health care [14], and willingness to seek help [15].

The studies described above focus on the recognition of depression, whereas less is known about age comparisons of mental health literacy for psychosis and other mental illnesses. The current study aims to address this by examining differences in various elements of mental health literacy in adults aged 18 years and older. Using an Australian national survey, the current study investigates age differences in the recognition of depression and schizophrenia, and the associated risk factors, treatments and sources of help. Although this investigation is exploratory, based on the results of previous studies we anticipate that the oldest (70+ years) and youngest (18–24 years) respondents will report lower levels of recognition of depression and schizophrenia, respectively, than respondents in other age groups.

## Methods

### Survey methodology

A national clustered household survey of Australian adults (18+ years) was conducted by the survey company AC Nielsen in 2003–2004. The survey methods have been described in detail elsewhere [16]. In brief, households were sampled from 250 census districts in all Australian states and territories (in both rural and metropolitan areas) and the resident from each household with the most recent birthday was interviewed. Of the 28,947 households visited, 14,630 were unable to be contacted, 7,815 refused, 1,132 were unavailable at the time of interview, 287 were not eligible, 383 did not speak English, 213 were incapable of responding, and 181 were unavailable for the entire duration of the survey. A final sample of 3,988 (41.2% males and 58.8% females) was achieved, with a response rate of 34%. Response rates were similar across states and territories (NSW: 38%; Qld: 33%; Vic: 28%; SA: 34%; WA: 34%; Tas: 39%), with the exception of the ACT and NT, in which response rates were higher (ACT: 50%; NT: 62%). Response rates were also consistent across age groups. Compared to population data, the sample had a lower representation of younger people and a higher representation of older people. Sample weighting was used to correct for this. Ethics approval for the study was granted by the Australian National University Human Research Ethics Committee.

### Survey interview

Respondents were randomly assigned to receive either a male ("John") or female ("Mary") version of one of four vignettes describing either: (1) depression, (2) early schizophrenia, (3) depression and suicidal thoughts, or (4) chronic schizophrenia. The symptoms described in the vignettes met both DSM-IV [17] and ICD-10 [18] diagnostic criteria for major depression or schizophrenia. For the current investigation, only data from vignettes (1) depression (n = 1001) and (2) early schizophrenia (n = 997) were analysed (Table 1). These portrayed a person at the threshold of a mental disorder and are most relevant to the issue of early recognition and intervention. Following presentation of the vignette, respondents were asked a number of questions to determine if they recognised the mental disorder depicted in the vignette, and to document their beliefs about the best kinds of help, treatments, and likely causes for the disorder. These questions were taken from a previous national survey of mental health literacy [1]. Although not relevant to the analyses reported here, the interview also included questions on the likely prognosis for the person described in the vignette, beliefs associated with stigma and discrimination, attitudes towards contact with people like those in the vignette, and the health and sociodemographic characteristics of the respondent.

**Table 1:** Case vignettes used in the survey.

Disorder	Vignette
Depression	John is 30 years old. He has been feeling unusually sad and miserable for the last few weeks. Even though he is tired all the time, he has trouble sleeping nearly every night. John doesn't feel like eating and has lost weight. He can't keep his mind on his work and puts off making decisions. Even day-to-day tasks seem too much for him. This has come to the attention of his boss, who is concerned about John's lowered productivity.
Early Schizophrenia	John is 24 and lives at home with his parents. He has had a few temporary jobs since finishing school but is now unemployed. Over the last six months he has stopped seeing his friends and has begun locking himself in his bedroom and refusing to eat with the family or to have a bath. His parents also hear him walking about his bedroom at night while they are in bed. Even though they know he is alone, they have heard him shouting and arguing as if someone else is there. When they try to encourage him to do more things, he whispers that he won't leave home because he is being spied upon by the neighbour. They realize he is not taking drugs because he never sees anyone or goes anywhere.

### Measures

To assess *knowledge and recognition of the disorder *shown in the vignette, respondents were asked the following open-ended question: 'What would you say, if anything, is wrong with John/Mary?'. Responses were coded into 11 categories and participants were considered correct if they received the depression vignette and answered 'depression' or they received the schizophrenia vignette and answered 'schizophrenia' or 'psychosis'. Where participant's responses did not fit one of the 11 specified categories, their responses were classified as 'other'. These responses were examined, and then coded into 5 categories: sleep-related issues (eg. lethargic, tired), problems with substance abuse, physical health problems (eg. diabetes), issues related to lifestyle (eg. diet, poor social life) and emotional or psychological issues (eg. poor self-esteem, trauma).

To assess *beliefs about sources of help*, respondents were asked 'How do you think John/Mary could best be helped?' Open ended responses were coded into the following categories: 'talk over with friends/family,' 'see a doctor/GP,' 'see a psychiatrist,' 'take medication,' 'see a counsellor or have counselling,' 'other' and 'don't know.' Responses that were classified as 'other' were examined separately and then coded into the following categories: 'further engagement with personal interests' (eg. join a club, get out more), 'seek additional professional help' (eg. group therapy, blood tests), and 'get others actively involved to help' (eg. colleagues, friends and family).

*Beliefs about treatment *were assessed by asking respondents to rate: (a) various treating professionals (eg. GP, psychologist), (b) various medications (eg. pain relievers, antidepressants), and (c) other treatments (eg. physical activity, psychotherapy, hypnosis) as either 'helpful, 'harmful' or 'neither.'

Causal beliefs about depression and psychosis were also assessed [19]. Respondents were asked to rate the likelihood of the following as *causes for the disorder described in the vignette*: 'virus or infection', 'allergies', 'day-to-day problems,' 'the recent death of a close friend or relative,' 'experiencing a recent trauma,' 'childhood problems,' 'genetics,' 'being a nervous person' and 'weakness of character.' Response categories included: 'very likely,' 'likely,' or 'not likely.'

### Statistical analysis

Data were pooled across male and female versions of the vignettes for analysis, as previous work showed no differences [1]. Age of respondents was classified into five groups: 18–24, 25–39, 40–54, 55–69, 70+ years and cross-tabulated with the variables of interest. Percentages were calculated using the Complex Samples procedure in SPSS 14.0, which takes into account sampling weights and geographic clustering of the sample. Response categories with frequencies of less than 2% for both vignettes were omitted from the analysis. Z-tests were used to assess differences between age groups, with a significance level set at p < .001. However, only significant group differences with a "medium" effect size equivalent to Cohen's h = 0.5 [20] are discussed. Other differences were statistically significant, but did not reach the minimum effect size of 0.5.

## Results

### Recognition of the disorder

Table 2 shows coded responses to the open-ended question 'What would you say, if anything, is wrong with John/Mary?' as a function of age and vignette type. Depression was the most common response among participants of all ages who received the depression vignette. Adults aged 70 years and above were less accurate than all other age groups in identifying the symptoms of depression; 18–24 years (z = 4.77, *h *= .64), 25–39 years (z = 5.76, *h *= .61), 40–54 years (z = 5.00, *h *= .53), and 55–69 years (z = 4.30, *h *= .50). However, those in the oldest age group (70+ years) were more likely than those in the youngest age group (18–24 years) to suggest that the person has a problem in general (z = 4.01, *h *= .53). For respondents who received the schizophrenia vignette, adults in the oldest age group (70+ years) were significantly poorer than middle aged adults (40–54 years) at correctly recognising the disorder (z = 4.72, *h *= .50). Younger adults (18–24 years) showed a greater tendency than older adults (70+ years) to misidentify schizophrenia as depression (z = 3.28, *h *= .50).

**Table 2:** Percentage of respondents endorsing each category to describe the problem shown in the vignette.

	Depression Vignette					Schizophrenia Vignette				
*Age*	18–24 *n *= 96	25–39 *n *= 293	40–54 *n *= 250	55–69 *n *= 213	70+ *n *= 149	18–24 *n *= 102	25–39 *n *= 246	40–54 *n *= 296	55–69 *n *= 215	70+ *n *= 138

Depression	**71.7**	**70.8**	**67.4**	**64.9**	**41.5**	**42.5**	**36.7**	**40.8**	**25.0**	**22.8**
Nervous breakdown	0.0	1.2	0.0	1.0	1.5	0.0	2.2	0.6	2.4	3.5
Schizophrenia	0.0	0.0	0.0	0.0	0.0	**42.7**	**40.9**	**46.9**	**42.6**	**24.1**
Mental illness	0.0	3.9	2.9	2.6	4.5	27.0	25.6	20.5	23.7	18.2
Psychological problem	0.7	4.5	5.4	5.0	5.1	7.4	9.3	15.1	14.8	18.1
Stress	*18.3*	*20.2*	*17.2*	*14.1*	*8.4*	2.0	2.3	2.1	5.8	4.0
Has a problem	**4.8**	**8.9**	**13.9**	**15.5**	**22.3**	7.4	9.3	8.6	6.8	13.8
Cancer	0.7	1.2	0.7	1.7	2.5	0.0	0.4	0.0	0.0	0.0
Other	43.9	38.8	42.1	44.4	47.8	32.2	38.1	34.8	40.5	43.8
Don't know	2.8	2.3	0.6	3.7	7.2	2.7	1.8	1.2	1.4	1.0

Note: Bolded results indicate a significant difference between age groups (p < .001) with a minimum effect size of Cohen's *h *= .50. Italicised results indicate a significant difference between age groups (p < .001), but without a minimum effect size of .50.

42.3% of respondents who received the depression vignette selected the category 'other', and this is reported in Table 2 as a function of age. The coded responses for these participants were as follows: 9.7% indicated a sleep-related difficulty, 2.6% indicated a problem related to drugs or alcohol, 24.8% indicated a physical health problem, 34.5% indicated a lifestyle issue, 24.6% indicated an emotional or other psychological issue, and 3.8% of responses were impossible to code. There were no differences within each response category across age groups. 37.7% of respondents who received the schizophrenia vignette selected the category 'other'. Of these respondents, 6.4% indicated a problem with drugs or alcohol, 54.6% indicated an emotional or psychological issue, 0.8% indicated a sleep-related problem, 2.1% indicated a physical health issue, 30.1% indicated a lifestyle issue, and 4.3% of responses were impossible to code. Only one significant age difference was identified: older adults (70+ years) were more likely than middle aged adults (40–54 years) to indicate that the problem described in the vignette was related to a lifestyle issue (z = 3.25, *h *= .54).

### Beliefs about the best kind of help

Table 3 shows the percentage of respondents identifying each help source as being the best for the person described in the vignette. For respondents who received the depression vignette, seeing a doctor or GP was rated by all age groups as the best kind of help. Compared with those aged over 70 years, family and friends were more likely to be endorsed by young adults (18–24 years) (z = 3.18, *h *= .50) and counselling was more likely to be endorsed by young (18–24 years) (z = 3.55, *h *= .50) and middle aged (40–54 years) (z = 4.52, *h *= .50) adults as the best kinds of help for depression.

**Table 3:** Percentages of respondents naming types of help as being the best for the person described in the vignette.

	Depression Vignette					Schizophrenia Vignette				
*Age*	18–24 *n *= 96	25–39 *n *= 293	40–54 *n *= 250	55–69 *n *= 213	70+ *n *= 149	18–24 *n *= 102	25–39 *n *= 246	40–54 *n *= 296	55–69 *n *= 215	70+ *n *= 138

Friends/Family	**35.6**	**23.6**	**23.1**	**18.4**	**14.9**	**38.0**	**23.9**	**18.7**	**15.3**	**18.2**
GP	51.4	57.4	58.2	53.5	58.4	22.4	34.1	36.1	34.5	24.7
Psychiatrist	16.9	9.2	10.7	14.3	21.4	29.0	29.8	33.8	34.8	31.0
Medication	6.8	6.1	8.3	4.4	2.6	3.6	9.6	8.8	7.0	10.9
Counselling	**35.4**	**30.0**	**33.3**	**19.8**	**13.7**	**35.2**	**33.3**	**29.1**	**23.8**	**15.1**
Other	34.5	40.4	37.7	39.9	32.4	34.9	40.3	39.8	45.2	37.8
Don't know	1.2	1.4	1.6	2.1	3.4	4.5	1.6	0.8	1.2	3.9

Note: Bolded results indicate a significant difference between age groups (p < .001) with a minimum effect size of Cohen's *h *= .50. Italicised results indicate a significant difference between age groups (p < .001), but without a minimum effect size of .50.

Young adults (18–24 years) who received the schizophrenia vignette were more likely than those aged 55–69 years (z = 4.14, *h *= .51) and over 70 years (z = 3.40, *h *= .50) to rate family and friends as the best kind of help, and more likely than those aged over 70 years to rate counselling as the best kind of help (z = 3.30, *h *= .50).

37.3% of respondents who received the depression vignette selected the category 'other', and this is reported in Table 3 as a function of age. The coded responses for these participants were as follows: 35.1% suggested that John/Mary would benefit from pursuing personal interests, 36.2% suggested seeking other additional help, 23.9% suggested that the active involvement of others would be beneficial, and 4.8% of responses were impossible to code. 39.5% of respondents who received the schizophrenia vignette selected the category 'other'. Of these respondents, 51.3% suggested seeking additional help, 21.6% indicated pursuing personal interests, 21.1% suggested the active involvement of others, and 6.1% of responses were impossible to code. For both vignettes, there were no differences within response categories across age groups.

### Beliefs about helpfulness of treatments

Table 4 shows the percentage of respondents who rated each treatment type (professionals, medical, lifestyle, psychological) for depression and schizophrenia as "helpful". For depression, respondents aged 18–24 years were more likely than those aged over 70 years to rate psychologists as helpful (z = 3.36, *h *= .50). Compared with adults aged over 70 years, 18–24 (z = 3.90, *h *= .54) and 25–39 year olds (z = 4.15, *h *= .50) were more likely to rate reading about the problems as helpful, 25–39 year olds were more likely to rate counsellors (z = 4.12, *h *= .50) as helpful, and 40–54 year olds more often rated naturopaths as helpful (z = 4.25, *h *= .50).

**Table 4:** Percentage of respondents rating various treatments as helpful for the person described in the vignette.

	Depression Vignette					Schizophrenia Vignette				
*Age*	18–24 *n *= 96	25–39 *n *= 293	40–54 *n *= 250	55–69 *n *= 213	70+ *n *= 149	18–24 *n *= 102	25–39 *n *= 246	40–54 *n *= 296	55–69 *n *= 215	70+ *n *= 138

GP	85.9	88.3	86.3	85.7	90.9	**64.8**	**76.5**	**85.4**	**70.6**	**77.4**
Pharmacist	31.8	32.0	34.5	40.2	42.1	14.9	22.8	22.1	29.3	29.0
Counsellor	**85.5**	**88.4**	**85.7**	**73.8**	**68.2**	**92.4**	**90.1**	**90.1**	**74.3**	**70.5**
Social worker	*66.4*	*69.4*	*61.4*	*51.6*	*61.9*	*70.9*	*73.0*	*72.3*	*62.5*	*54.9*
Phone counselling	*73.0*	*71.8*	*61.4*	*52.4*	*54.7*	**62.6**	**62.8**	**60.1**	**50.0**	**38.0**
Psychiatrist	65.1	69.8	68.0	56.7	58.9	*76.9*	*84.9*	*85.0*	*76.5*	*69.7*
Psychologist	**74.3**	**72.0**	**72.9**	**54.6**	**52.2**	**71.5**	**79.3**	**80.4**	**68.1**	**55.1**
Close family	74.8	71.2	67.4	61.7	62.9	68.8	68.0	60.7	57.5	57.3
Close friends	84.9	81.1	78.8	71.6	72.5	*84.7*	*71.8*	*73.0*	*67.8*	*71.1*
Naturopath/herbalist	**28.5**	**37.1**	**44.9**	**28.3**	**23.7**	22.3	26.8	26.0	19.8	18.9
Clergy	37.2	46.1	43.3	46.6	54.0	29.3	31.1	41.8	42.2	40.1
Vitamins, minerals	53.5	51.5	54.4	43.6	44.6	31.3	35.0	30.9	27.6	29.5
Antidepressants	*50.5*	*51.9*	*46.4*	*45.9*	*32.2*	47.5	49.2	53.0	52.9	42.2
Antibiotics	11.0	10.7	8.0	11.0	13.6	4.1	6.5	1.4	2.6	6.6
Sleeping pills	30.2	27.5	16.6	21.8	27.6	22.3	17.3	20.0	13.4	18.2
Antipsychotics	12.7	11.8	13.4	11.8	3.1	*29.4*	*36.6*	*38.0*	*31.7*	*19.5*
Physical activity	92.1	94.2	91.2	89.8	91.7	91.4	88.5	85.2	86.1	88.2
Read about problem	**87.7**	**86.3**	**76.8**	**74.3**	**66.2**	**88.8**	**84.9**	**84.0**	**67.1**	**66.4**
Get out more	86.8	86.1	86.6	88.2	88.9	94.2	87.1	85.2	84.7	87.9
Learn relaxation	85.0	87.3	84.0	81.2	76.1	80.7	75.8	79.0	74.7	75.2
Cut out alcohol	49.0	58.4	53.8	58.2	58.9	58.3	70.2	68.8	63.8	62.2
Psychotherapy	*44.9*	*49.0*	*48.1*	*37.8*	*31.5*	61.6	62.6	63.2	53.0	48.5
Hypnosis	20.8	22.4	25.6	18.6	22.8	26.9	32.1	31.3	32.0	21.5
Psychiatric ward	16.1	16.7	16.9	17.7	12.8	*27.0*	*33.5*	*35.2*	*36.1*	*19.4*
Occasional drink	***37.3***	***36.3***	***47.2***	***50.5***	***56.8***	27.2	27.0	29.0	35.9	42.4
Special diet	45.9	45.3	54.0	49.9	43.2	*29.9*	*42.1*	*47.7*	*42.9*	*39.8*

Note: Bolded results indicate a significant difference between age groups (p < .001) with a minimum effect size of Cohen's *h *= .50. Italicised results indicate a significant difference between age groups (p < .001), but without a minimum effect size of .50.

For schizophrenia, older adults (40–54 years) were more likely than younger people (18–24 years) to endorse seeing a GP as helpful (z = 3.77, *h *= .50). Compared with adults aged over 70 years, 18–24 year olds were more likely to rate seeing a counsellor (z = 4.74, *h *= .58) and receiving telephone counselling (z = 3.84, *h *= .50) as helpful, and 25–39 year olds (z = 5.07, *h *= .51) and 40–54 year olds (z = 5.37, *h *= .54) were more likely to rate psychologists as helpful. Younger adults (18–24 years) were more likely than 55–69 year olds (z = 4.58, *h *= .54) and adults aged over 70 years (z = 4.23, *h *= .56) to rate reading about the problem as helpful.

### Beliefs about likely causes

Table 5 shows percentages of respondents who rated various causes for depression and schizophrenia as either "very likely" or "likely". No age differences were found in respondents for the depression vignette. Adults aged over 70 years more often rated weakness of character as a cause for schizophrenia than adults aged 55–69 years (z = 3.86, *h *= .50).

**Table 5:** Percentages of respondents rating likely causes for the disorder described in the vignette.

	Depression Vignette					Schizophrenia Vignette				
*Age*	18–24 *n *= 96	25–39 *n *= 293	40–54 *n *= 250	55–69 *n *= 213	70+ *n *= 149	18–24 *n *= 102	25–39 *n *= 246	40–54 *n *= 296	55–69 *n *= 215	70+ *n *= 138

Virus or infection	45.1	50.9	50.0	52.0	58.8	37.5	31.8	34.0	31.5	38.8
Allergy	40.6	45.6	43.8	49.4	55.5	24.0	34.1	33.5	35.5	37.1
Day-to-day problems	97.9	97.7	96.8	97.0	96.8	97.4	88.0	91.4	88.0	87.6
Recent death of close friend/relative	97.7	97.1	97.0	96.2	91.8	*95.0*	*88.3*	*88.6*	*85.4*	*80.9*
Recent trauma	89.5	93.5	96.7	95.6	93.1	90.6	85.8	88.9	87.3	81.3
Childhood problems	93.9	93.1	92.7	92.5	87.6	*97.1*	*92.6*	*90.5*	*91.0*	*85.6*
Genetic	60.1	75.0	69.7	68.3	71.8	68.8	73.9	77.2	68.5	74.6
Nervousness	63.6	68.4	68.8	71.6	71.1	58.9	60.1	59.9	59.7	66.0
Weakness of character	*42.2*	*41.7*	*42.9*	*42.4*	*57.4*	**44.9**	**37.9**	**38.4**	**35.1**	**57.5**

Note: Bolded results indicate a significant difference between age groups (p < .001) with a minimum effect size of Cohen's *h *= .50. Italicised results indicate a significant difference between age groups (p < .001), but without a minimum effect size of .50.

## Discussion

The results suggest that in the Australian community there is variation in mental health literacy across age groups. There were age differences identified in the ability to recognise depression and psychosis, and in preferences for the sources of treatment and for treatment providers.

The age differences occurred primarily between the oldest age group and all other ages. In particular, respondents in the oldest age group (70+) were less likely to correctly identify the mental illness described in the vignette, endorsed fewer sources of treatment as helpful, and were more likely to believe that schizophrenia was related to character weakness. These findings are consistent with previous research indicating poorer mental health literacy in older age groups [11,12]. Poorer mental health literacy amongst older people may due to a number of factors. Messages about mental health may be predominantly delivered in media less commonly accessed by the elderly (such as the Internet). School education programs are a primary source of mental health literacy for younger people, but such programs were not systematically available in earlier decades. Current mental health literacy campaigns may not be targeted towards older adults. Stigma among older people may be greater [21], and inhibit openness to new information about the recognition and aetiology of mental disorders.

The results also indicate that younger adults view informal sources of help more favourably than older adults, such as talking to friends or family. Younger people are also more likely than older age groups to consider consulting books as helpful. Counselling and counsellors appeal to younger and younger-middle aged adults, possibly because they understand the role of the counsellor more fully than that of a psychiatrist or psychologist.

Younger people have better mental health literacy concerning depression than schizophrenia. Although the 18–24 years age group were superior at correctly identifying depression, they were less competent at correctly identifying schizophrenia, and were more likely to misdiagnose schizophrenia as depression. The cause of this misidentification is not known, but it may reflect the predominant emphasis on depression in Australia's public health programs. Younger people also continue to rate informal, non-biologically-based sources of treatment for schizophrenia favourably, and are less likely to endorse treatments recommended by clinical practice guidelines, such as seeing a psychiatrist and receiving medication [22]. This is especially problematic in conjunction with the overall lack of knowledge about schizophrenia found in this population.

Limitations of the present study should be acknowledged. Firstly, given that multiple comparisons were made, there is the possibility of detecting spuriously significant differences, despite the use of conservative significance level and effect size cut-offs. Secondly, as the data are cross-sectional, it is not possible to determine whether the age differences found genuinely reflect changes with age or whether they are due to a cohort effect. Another limitation is that both vignettes portrayed a relatively young person. Thus, the elderly were responding about the helpfulness of interventions for someone younger, rather than someone their own age. A final limitation is the number of people who refused to participate in the study, introducing the possibility of biases in the estimated population parameters.

## Conclusion

There are age differences in the Australian community regarding mental health literacy for depression and schizophrenia. While younger people have more accurate knowledge than the elderly surrounding the recognition and treatment of depression, their mental health literacy concerning schizophrenia is not as proficient as their depression literacy. Younger people also tend to identify informal treatment sources as helpful, while the elderly rate fewer sources as helpful overall. They are more likely to attribute the development of schizophrenia to personal characteristics. These findings suggest that consideration should be given to developing targeted programs for increasing older people's mental health literacy for depression. Specific strategies should involve messages about depression that appeal to the unique interests and needs of this age group, and perhaps their pre-existing belief systems [23]. Younger people may benefit from campaigns that emphasise information about the nature of schizophrenia and its appropriate treatments. Improving the mental health literacy of today's young people could potentially result in a generational shift in attitudes and knowledge about mental disorders.

## Declaration of competing interests

The author(s) declare that they have no competing interests.

## Authors' contributions

LF carried out the data analysis and drafted sections of the manuscript. LL assisted in data analysis and helped draft the manuscript. KG, HC and AJ conceived and designed the study and assisted in the preparation of the manuscript. All authors have read and approved the final manuscript.

## Pre-publication history

The pre-publication history for this paper can be accessed here:


